# Steroid hormone analysis in diagnosis and treatment of DSD: position paper of EU COST Action BM 1303 ‘DSDnet’

**DOI:** 10.1530/EJE-16-0953

**Published:** 2017-02-20

**Authors:** A Kulle, N Krone, P M Holterhus, G Schuler, R F Greaves, A Juul, Y B de Rijke, M F Hartmann, A Saba, O Hiort, S A Wudy, on behalf of the EU COST Action

**Affiliations:** 1 Division of Pediatric Endocrinology and DiabetesDepartment of Pediatrics, Christian-Albrechts-University, Kiel, Germany; 2 Academic Unit of Child HealthDepartment of Oncology and Metabolism, University of Sheffield, Sheffield, UK; 3 Veterinary Clinic for ObstetricsGynecology and Andrology of Large and Small Animals, Justus-Liebig-University, Giessen, Germany; 4 School of Health and Biomedical SciencesRMIT University, Victoria, Australia; 5 Department of Growth and ReproductionRigshospitalet, University of Copenhagen, Copenhagen, Denmark; 6 Department of Clinical ChemistryErasmus Medical Center, Rotterdam, Netherlands; 7 Department of SurgicalMedical and Molecular Pathology and Critical Care Medicine, University of Pisa, Pisa, Italy; 8 Pediatric Endocrinology and DiabetologyChildren’s Hospital, University of Luebeck, Luebeck, Germany; 9 Steroid Research & Mass Spectrometry UnitLaboratory for Translational Hormone Analytics, Division of Pediatric Endocrinology and Diabetology, Center of Child and Adolescent Medicine, Justus-Liebig-University, Giessen, Germany

## Abstract

Disorders or differences in sex development (DSD) comprise a heterogeneous group of conditions with an atypical sex development. For optimal diagnosis, highly specialised laboratory analyses are required across European countries. Working group 3 of EU COST (European Cooperation in Science and Technology) Action BM 1303 ‘DSDnet’ ‘Harmonisation of Laboratory Assessment’ has developed recommendations on laboratory assessment for DSD regarding the use of technologies and analytes to be investigated. This position paper on steroid hormone analysis in diagnosis and treatment of DSD was compiled by a group of specialists in DSD and/or hormonal analysis, either from participating European countries or international partner countries. The topics discussed comprised analytical methods (immunoassay/mass spectrometry-based methods), matrices (urine/serum/saliva) and harmonisation of laboratory tests. The following positions were agreed upon: support of the appropriate use of immunoassay- and mass spectrometry-based methods for diagnosis and monitoring of DSD. Serum/plasma and urine are established matrices for analysis. Laboratories performing analyses for DSD need to operate within a quality framework and actively engage in harmonisation processes so that results and their interpretation are the same irrespective of the laboratory they are performed in. Participation in activities of peer comparison such as sample exchange or when available subscribing to a relevant external quality assurance program should be achieved. The ultimate aim of the guidelines is the implementation of clinical standards for diagnosis and appropriate treatment of DSD to achieve the best outcome for patients, no matter where patients are investigated or managed.

## Introduction

Disorders or differences in sex development (DSD) comprise a heterogeneous group of conditions with an atypical sex development. Patients with DSD are complex and rare, and multi-disciplinary teams are needed for optimal diagnosis and management. Thus, highly specialised laboratory analyses are required across European countries. COST (European Cooperation in Science and Technology) Action ‘DSDnet’ forms a network bringing together different stakeholders and people interested in DSD, scientists, clinicians as well as people with DSD (www.dsdnet.eu). Working group 3 ‘Harmonisation of Laboratory Assessment’ of the COST action ‘DSDnet’ has developed recommendations on laboratory assessment for DSD regarding the use of technologies and analytes to be investigated. This important work will form the basis of future European reference network for rare endocrine disorders. This position paper on steroid hormone analysis in diagnosis and treatment of DSD was compiled by a group of specialists in DSD and/or hormonal analysis, either from participating European countries or international partner countries.

### Relevance of clinical steroid analysis

The Chicago consensus statement and recent literature regarding disorders of sex development emphasises the need for comprehensive diagnosis and treatment of DSD (1, 2, 3, 4, 5). Diagnosis of DSD remains challenging for involved paediatricians, endocrinologists, geneticists, urologists and other related disciplines, as even with novel approaches a specific molecular diagnosis is only achieved in about 30–50% of patients with 46,XY DSD (4). Thus, the diagnostic pathway in patients with DSD requires close interlinking between the clinical, biochemical and genetic diagnostic work-up.

**Figure 1:**
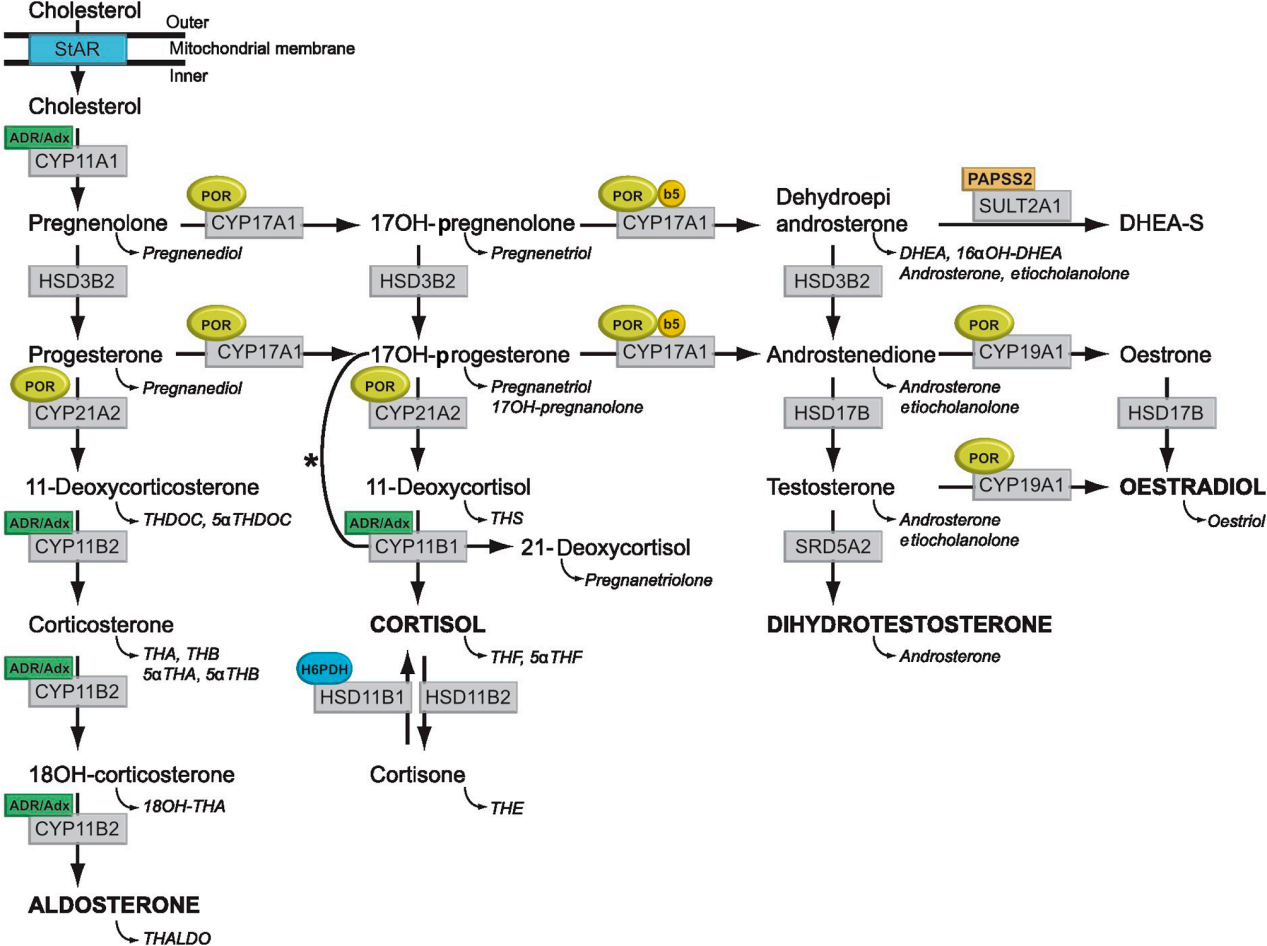
Synthesis and metabolism of hormonal steroids. This figure illustrates the formation of the major hormone classes from cholesterol. Steroid names in conventional script are steroid hormones and precursors; those in italics are urinary metabolites of the aforementioned. The major transformative enzymes are in rectangular boxes, the cofactor (‘facilitator’) enzymes in ovals. Mitochondrial CYP type I enzymes requiring electron transfer via adrenodoxin reductase (ADR) and adrenodoxin (Adx) CYP11A1, CYP11B1 and CYP11B2 are marked with a labelled box ADR/Adx. Microsomal CYP type II enzymes receive electrons from P450 oxidoreductase (POR), CYP17A1, CYP21A2, CYP19A1 and are marked by circled POR. The 17,20-lyase reaction catalysed by CYP17A1 requires in addition to POR also cytochrome b5 indicated by a circled b5. Similarly, hexose-6-phosphate dehydrogenase (H6PDH) is the cofactor-generating enzyme for 11β-HSD1 (HSD11B1). The asterisk (*) indicates the 11-hydroxylation of 17-hydroxyprogesterone to 21-deoxycortisol in 21-hydroxylase deficiency. The conversion of androstenedione to testosterone is catalysed by HSD17B3 in the gonad and AKR1C3 (HSD17B5) in the adrenal. CYP11A1, P450side-chain cleavage enzyme; CYP11B1, 11β-hydroxylase; CYP11B2, aldosteronesynthase; CYP17A1, 17α-hydroxylase/17,20-lyase; CYP21A2, 21-hydroxylase; HSD3B2, 3β-hydroxysteroid dehydrogenase type 2; HSD11B1, 11β-hydroxysteroid dehydrogenase type 1; HSD11B2, 11β-hydroxysteroid dehydrogenase type 2; HSD17B, 17β-hydroxysteroid dehydrogenase; PAPSS2, 3′-phosphoadenosine 5′-phosphosulfate synthase 2; SRD5A2, 5α-reductase type 2; StAR, steroidogenic acute regulatory protein; SULT2A1, sulfotransferase 2A1.

Traditionally, hormonal analyses in blood or urine have been used as part of the first-line diagnostic approach (Fig. 1). In current practice, this is more frequently used in combination with molecular genetic analyses. In addition to diagnostic data, biochemical analysis will provide additional functional information guiding further management, disease monitoring and explain differences in phenotypic expression. However, this heavily depends on local and national diagnostic pathways and regional differences in accessibility to highly specialised analyses. There are significant differences in funding streams, clinical and laboratory resources as well as the interplay with research laboratories, resulting in a heterogeneous situation in Europe. In many countries, clinical endocrinology has increasingly become dominated by economic constraints. Although hormone measurement has traditionally been a mainstay of clinical endocrinology, the absorption of hormone labs by centralised laboratory units is associated with the risk of loss of expertise in hormone test development, selection and data interpretation. A Pan-European and ultimately a global approach should aim for a harmonisation of diagnostic pathways according to requirements achieving the correct diagnosis.

The majority of 46,XX infants presenting with virilisation of the external genitalia will have congenital adrenal hyperplasia (CAH). In such cases, a quantification of multiple steroids in a steroid profile is very important to detect rare forms of steroid biosynthesis disorders. A recent report on exome sequencing exemplifies the significant benefit of next-generation sequencing techniques for diagnosing 46,XY DSD (4). However, the paper indirectly illustrates the requirement of detailed clinical and comprehensive biochemical data for a holistic understanding of individual DSD cases.

Hormonal analysis (6) is not only important for the initial diagnosis of DSD. It also remains a decisive corner stone for monitoring adequate hormone replacement in various conditions with the goal of avoiding adrenal crises, ensuring optimal development of growth, weight and puberty, supporting sexual function and optimising quality of life in patients with DSD. It is of paramount importance that any meaningful hormone data interpretation in relation to DSD has to take into consideration the patient’s individual clinical picture and requires age- and sex-specific reference intervals due to the changing physiology of the developing child and young person (7, 8, 9, 10).

### Advantages of clinical steroid analysis

Clinical steroid profiling remains an important first-line approach to the diagnosis of DSD, as it provides fast and comprehensive results and thus allows for a rapid differential diagnostic orientation. In cases of CAH, it has a good phenotype–genotype correlation (11).

### Challenges of clinical steroid analysis

Over the course of recent years, it has become increasingly difficult to recruit healthy volunteers in childhood and adolescence to establish normative reference data from a control cohort. This is mainly the result of ethical concerns and prevents the implementation of accurate age- and sex-specific reference intervals. Soeborg *et al*. (10) emphasise that medical treatment, such as exogenous steroids, hepatic metabolism-interacting agents or liquorice-containing sweets can influence steroid metabolism and may influence the interpretation of results (12). Recent studies also describe an impact of nutritional status on steroid hormone concentrations (13). This indicates that there is an ongoing need for establishing reference intervals. However, through the harmonisation of laboratory tests, there is the potential to develop common reference intervals. The work required to generate these data should be shared thus allowing results from different laboratories to be directly compared.

## Analytical methods

The main aim of clinical guidelines is to implement clinical standards for diagnosis and appropriate treatment to achieve the best outcome for patients, no matter where patients are investigated or managed. Therefore, all methods require appropriate validation to ensure that they are fit for their intended clinical purpose (14). This includes the important peer comparison processes of sample exchange or, if available, participation in an external quality assurance scheme (EQA).

### Immunoassay methods

The principle of all immunoassay-based methods is the binding of an antigen to an antibody. In the late 1970s, the radioligands were replaced with chemiluminescence, enzymatic or fluorescent ligands. In general, immunoassay-based methods for steroid measurements detect a tracer rather than directly the analyte. Most clinical biochemistry laboratories adopted these assays due to their low cost, simplicity and fast turn-around times. Immunoassays are available for various steroidal analytes as commercial kits on automated platforms. One advantage of radio immunoassay (RIA) methods is the wide and extensive experience as these assays have been used for almost 50 years in clinical routine and research laboratories. During this time, a considerable amount of data have been accumulated, and numerous studies have enriched the field of endocrinology (15). For some analytes such as estradiol, very sensitive techniques exist (16, 17). Thus, immunoassays can produce highly specific results, particularly in combination with preceding extraction and/or chromatography of the samples (18). Improved separation through extraction is particularly important for newborns, especially if born early, as the fetal adrenal zone that produces a different mix of steroids persists until at least the equivalent of term (19).

However, numerous commercial assays, especially automated immunoassays, have recently been shown to have impaired specificity due to cross-reactivity of the antibody and other unidentified interferences from the matrix (20, 21). Therefore, tests evaluated in serum/plasma from healthy adults do not necessarily produce reliable values in neonates or pregnant women (22) or when applied in different matrices such as saliva or urine. The rapid analysis time in many high-throughput analytical platforms may also be observed at the expense of poorer sensitivity (detection limit). Furthermore, immunoassays can only measure one steroid per analysis. Thus, larger volumes of serum/plasma are required for analysis. This can be particularly challenging when measuring small-volume samples from newborns and infants. The use of radioactivity in RIAs requires special laboratory facilities and generates radioactive waste. The lack of standardisation of immunoassays represents a major problem for the comparability of laboratory results, and in many cases, method-specific reference intervals must be considered for interpretation. In the current European landscape, immunoassays are still commonly employed for steroid hormone analysis. However, in the light of future developments, it can be expected that immunoassays will increasingly be replaced by mass spectrometry-based methods.

### Mass spectrometry-based methods

Mass spectrometry-based steroid hormone assays are physicochemical analytical techniques identifying the analyte by determining typical mass-to-charge ratios of the respective molecule or its typical fragments. In contrast to conventional isotopic and non-isotopic immunoassay techniques, mass spectrometry allows for higher specificity (23). Liquid chromatography linked with tandem mass spectrometry (LC–MS/MS) enables targeted steroid hormone analysis of multiple analytes from a single sample (9, 10, 24, 25, 26, 27). Gas chromatography coupled with mass spectrometry (GC–MS) allows for the simultaneous determination of steroid hormones and metabolites within targeted as well as non-targeted approaches.

At first glance, mass spectrometry-based methods may appear rather costly due to the price of sophisticated instrumentation, maintenance of equipment and the need for qualified personal operating the instruments. However, in comparison to other diagnostic procedures, such as molecular genetics, imaging procedures (computed tomography, magnetic resonance imaging and isotope-based imaging techniques) or multiple immunoassays, hormone profiling by mass spectrometry is actually very cheap. The use of LC–MS/MS for steroid analysis is still challenging. One of the main complicating factors in mass spectrometric steroid analysis is the presence of isobaric interferences caused by ions of identical mass-to-charge ratio and similar fragmentation patterns (28). Although steroid analysis by LC–MS/MS is becoming increasingly available for routine use, validation and quality control present important future challenges (29). Reference intervals are not widely available and in contrast to earlier anticipation these are considerably dependent on individual specific laboratory settings, such as sample work-up and/or instrumentation.

Position 1: Although mass spectrometry is purported to be a superior technique, it is not available for all hormones and is currently not a realistic analytical option in all regions of Europe. Our position is therefore to support the appropriate use of both immunoassay and mass spectrometry-based methods for the diagnosis and monitoring of DSD. It is essential that clinicians should also know the characteristics and limitations of analytical methods used.

## Analytical matrices

Blood (serum vs plasma), urine and saliva (30, 31) are the biomaterials (i.e. matrices) most commonly used for clinical steroid hormone analysis. Saliva is less broadly established, and few studies on steroids analysed in saliva exist for the differential diagnosis of DSD, and the working group does not have a stance for or against the inclusion of this matrix currently. It is recommended that clinicians contact their laboratories in advance to follow their recommendations regarding appropriate type of sample as well as mode of shipment (32, 33, 34).

Position 2: Both, serum/plasma and urine, are established matrices for analysis for steroids and dependent on the specific DSD condition under consideration, analysis of steroids in either matrix may be appropriate.

## Harmonisation of laboratory tests

Initiatives in laboratory medicine that support harmonisation stem from Europe and are now being embraced globally. Bias, imprecision and interferences can all lead to erroneous results. As such, method validation is fundamental in establishing the extent and acceptability of each of these studies for clinical diagnostic assays to ensure that they operate within an accepted quality framework. Although each of these validation parameters is important, minimisation of bias is essential for harmonisation.

Harmonisation and, where practical, standardisation with traceability, are enormous challenges (35). The process has been described in terms of five supporting pillars, these are aimed at establishing: (1) certified reference materials (CRM); (2) reference measurement procedures (RMP); (3) reference laboratories; (4) participation in an EQA program and (5) reference intervals and decision limits (36).

In principle, full standardisation with traceability should be achievable for all steroids as they are small compounds of defined molecular weight. The Joint Committee for Traceability in Laboratory Medicine (JCTLM) was established in 2002 to support this process worldwide through the development of a database to recognise primary reference materials, methods and laboratories (www.bipm.org/jctlm. Accessed 19th June 2016). This JCTLM database, which is hosted by the Bureau International of Weights and Measures (BIPM) (Sevres Cedex, France), currently lists some (e.g. serum cortisol, oestradiol, progesterone and testosterone) but not all steroids important for the assessment of DSD (e.g. serum 17-hydroxyprogesterone, androstenedione, cortisone and dihydrotestosterone).

Participation in an EQA program is generally recognised to be the central pillar as it provides the framework for objective comparison of the result obtained by many laboratories for the one sample (37, 38). However, to proceed down this pathway for the harmonisation of laboratory assessment, we first need to establish a collaborative agreement on the analytes and their matrices that should be measured for the differential diagnosis of a DSD. Recently, a first EQA program for the harmonisation of serum dihydrotestosterone analysis has been launched (39).

Position 3: Laboratories should aim to participate in activities of peer comparison such as a sample exchange or preferably when available subscribe to a relevant external quality assurance program.

## Steroid analysis in conditions associated with DSD

### 46,XX DSD conditions

#### 21-Hydroxylase deficiency

CAH due to 21-hydroxylase deficiency (21OHD) is the most common cause of DSD in 46,XX individuals (40). Due to overproduction of androgens, 46,XX individuals usually present with ambiguous genitalia without palpable gonads. The condition is treated with glucocorticoids and if required mineralocorticoids. In serum/plasma, 17-hydroxyprogesterone and 21-deoxycortisol with or without ACTH stimulation (adrenocorticotropic hormone) are the indicative diagnostic parameters for 21OHD. Usually, no ACTH stimulation is required in classic CAH. Urinary steroid profile analysis, a non-invasive means, also allows for definitive diagnosis: 17-hydroxypregnanolone, pregnanetriol and pregnanetriolone are the key diagnostic urinary metabolites (41). Steroid monitoring of 21OHD is performed by the determination of the previously mentioned hormones and their metabolites in either serum, plasma, urine or saliva (42).

#### 11β-Hydroxylase deficiency

It is characterised by elevated serum/plasma 11-deoxycortisol and deoxycorticosterone. Individuals with 46,XX DSD suffering from 11β-hydroxylase deficiency also present with virilisation of the external genitalia. The onset of hyporeninemic, hypokalaemic hypertension is variable. The urinary steroid profile is dominated by elevated tetrahydro-11-deoxycortisol (43).

#### 3β-Hydroxysteroid dehydrogenase deficiency (3βHSDD)

This condition is clinically characterised by undermasculinisation in 46,XY individuals and virilisation in 46,XX individuals (44). The pathognomonic hormonal pattern is characterised by the elevation of 17-hydroxypregnenolone, dehydroepiandrosterone (DHEA) and dehydroepiandrosterone sulphate (DHEAS) in serum/plasma and increased excretion rates of their corresponding urinary metabolites including androstenetriol.

#### Cytochrome P450 (P450) oxidoreductase deficiency

This condition presents biochemically as combined 17-hydroxylase/lyase and 21-hydroxylase deficiency. It is caused by a defect of the electron-donating protein to microsomal P450 type 2 enzymes. The typical presentation is a child with ambiguous genitalia and Antley–Bixler syndrome; however, a wide spectrum of clinical presentations has been described (45).

### 46,XY-DSD conditions

#### Steroid acute regulatory protein (StAR) deficiency and P450 side chain cleavage enzyme (P450scc) deficiency

StAR deficiency leads to lipoid CAH, whereas deficiency of P450scc commonly leads to small adrenals. Both conditions present with similar clinical appearance. Only a few patients might have a severe salt loss crisis in the first months of life. The majority of 46,XY individuals show undervirilisation or complete feminisation. In 46,XX patients, no further clinical features might be present in the first months of life. Typically, glucocorticoids, mineralocorticoids and sex steroids are all low to undetectable. Treatment with glucocorticoids (e.g. hydrocortisone) and later with sex hormones should be monitored by determining the respective steroids (46).

#### 17-Hydroxylase/17,20 lyase deficiency

Typically, 17-deoxygenated steroids, e.g. corticosterone, are elevated, whereas 17-oxygenated steroids, such as cortisol and sex steroids, are markedly reduced or absent in serum/plasma. Urinary steroid profile analysis likewise reflects an increase in 17-deoxygenated over 17-oxygenated metabolites (47). Patients are clinically often glucocorticoid replete despite impaired cortisol synthesis as corticosterone excess with its glucocorticoid action is compensating for the lack of cortisol. The deficiency of sex hormone biosynthesis is causing DSD in these individuals. All affected individuals (46,XY and 46,XX) with complete deficiency classically fail to develop secondary sexual characteristics. Similar to 11β-hydroxylase deficiency, the onset of hyporeninemic, hypokalaemic hypertension is highly variable.

#### Cytochrome P450 (P450) oxidoreductase deficiency

See section on 46,XX DSD conditions.

#### 3βHSDD

See section on 46,XX DSD conditions.

#### 5α-Reductase deficiency

Patients with mutations in the 5α-reductase type 2 gene usually show undermasculinisation at birth. Usually there is some degree of masculinisation at puberty due to increasing concentrations of testosterone. Diagnosis can be established using the ratio of testosterone to dihydrotestosterone in serum/plasma before and after hCG (human chorionic gonadotropin) stimulation (48, 49). The diagnosis can also be made by assessing the ratio between 5α- and 5β-reduced steroids in the urinary steroid profile (50).

#### 17β-Hydroxysteroid dehydrogenase type 3 deficiency

Due to the lack of testosterone during male sex differentiation, 46,XY children are often born with almost female-appearing external genitalia (51). In serum/plasma, the ratio of androstenedione/testosterone after hCG stimulation is elevated. Urine steroid metabolomic profiling for this enzyme deficiency might not always be indicative for this gonadal enzyme defect.

Position 4: Harmonisation of the primary analytes for analysis of specific DSD is required. We propose that laboratories measure the diagnostic key steroids mentioned as described previously.

## Conclusions

As DSD represents a very heterogeneous and highly complex group of conditions, the integration of clinical, biochemical and genetic diagnostic approaches is required. The knowledge of steroid hormone biosynthesis is vital in understanding the pathogenesis of the specific condition. Furthermore, monitoring strategies of these entities are to a great extent based on biochemical parameters. Therefore, only most reliable methods are required. To achieve this aim, laboratories performing analyses for DSD need to operate within a quality framework and actively engage in harmonisation processes so that results and their interpretation are the same irrespective of the laboratory they are performed in.

However, the situation with respect to access to analytical technologies is very heterogeneous within Europe. This is due to essential differences in health care systems, modes of payment, different economical coverage and structure of biochemical services. This compromises the development of common strategies for Pan-European diagnosis and follow-up in DSD.

There is an urgent demand for establishing a network of highly specialised endocrine reference laboratories with expertise in DSD. These centres should have the required knowledge of analytical techniques, should have age- and sex-specific reference intervals to provide normative data and should provide experience with proper interpretation of values. It is essential to maintain and support existing laboratories with expertise in DSD. Therefore, the area of DSD also holds a deep political dimension. Interested parties are encouraged to contact head of working group 3 for setting up a network of suitable reference laboratories. Investments to create and maintain such reference centres of expertise are vital to achieving a Pan-European and ultimately a global landscape ensuring access to optimal laboratory assessment for DSD.

## Declaration of interest

The authors declare that there is no conflict of interest that could be perceived as prejudicing the impartiality of the research reported.

## Funding

O H (chair of COST action), S A W (chair of working group 3) and all further members of working group 3 (A K (co-chair), N K, P M H, G S, A J, Y B R, M F H, A S) as well as R F G (international partner) appreciate support from BMBS COST Action BM1303.

## Author contribution statement

All the authors have accepted responsibility for the entire content of this submitted manuscript and approved submission.
